# A randomized controlled trial to test the efficacy of trans-tympanic injections of a sodium thiosulfate gel to prevent cisplatin-induced ototoxicity in patients with head and neck cancer

**DOI:** 10.1186/s40463-019-0327-x

**Published:** 2019-01-16

**Authors:** Viannique Rolland, François Meyer, Matthieu J. Guitton, Richard Bussières, Daniel Philippon, Isabelle Bairati, Mathieu Leclerc, Mathieu Côté

**Affiliations:** 1 Department of Ophthalmology and Otorhinolaryngology, Head and Neck Surgery, Faculty of Medicine, Laval University, Pavillon Ferdinand-Vandry, Bureau 4889, 1050, avenue de la Médecine, Québec City, QC G1V A06 Canada; 2 Centre Hospitalier Universitaire de Québec, Hôtel-Dieu de Québec, Quebec City, QC Canada; 3 Department of Social and Preventive Medicine, Faculty of Medicine, Laval University, Quebec City, QC Canada; 4 Laval University Cancer Research Center, Quebec City, Canada; 5 CERVO Brain Research Center, Quebec City, QC Canada; 6 Department of Surgery, Faculty of Medicine, Laval University, Quebec City, QC Canada; 7 Department of Medicine, Faculty of Medicine, Laval University, Quebec City, QC Canada

**Keywords:** Head and neck cancer, Cisplatin, Ototoxicity, Trans-tympanic injection, Sodium thiosulfate, Quality of life

## Abstract

**Background:**

Cisplatin-induced hearing loss is frequent and severe. Antioxidants such as sodium thiosulfate (STS) can neutralize the effects of cisplatin. The objective of the trial was to test the efficacy of trans-tympanic injections of a STS gel to prevent cisplatin-induced ototoxicity.

**Methods:**

Eligible participants were newly diagnosed patients with stage III or IV squamous cell carcinoma of the mouth, oropharynx, hypopharynx, or larynx and scheduled to be treated by concurrent chemoradiation (CCR). Patients with asymmetric hearing were not eligible. The planed treatment included cisplatin 100 mg/m^2^ at days 1, 22 and 43. A baseline pre-treatment complete audiometric evaluation (pure tone at frequencies ranging from 0.5 to 14 kHz, bone conduction at 0.5–4 kHz and DPOAEs) was performed. Adverse effects were noted according to CTCAE.

On the day before the beginning of CCR, eligible and consenting patients were randomized to receive a trans-tympanic injection of the gel either in the left ear or in the right ear. A final post-treatment complete audiometric evaluation was scheduled to be performed 1 month after the end of CCR by audiologists kept blind to the ear assignment.

For the main outcome, the permanent threshold shift (PTS) in decibel (dB) was calculated as the difference between the final and baseline measures at all pure tone frequencies at 0.5–14 kHz for each patient and for each ear. The main outcome was assessed blindly in a mixed linear model with the PTS as the dependent variable and intervention, frequency, their interaction and radiation dose to the cochlea as independent variables.

**Results:**

Between January 2015 and April 2016, 13 patients were randomized. The trial was stopped in June 2016 for poor accrual. The average loss of hearing over all frequencies was 1.3 dB less for treated ears compared to control ears. Although not statistically (*p* = 0.61) nor clinically significant, the difference was in favor of the treated ears for all frequencies between 3 and 10 kHz.

**Conclusions:**

Our trial suggests that STS deposited on the round window was safe for the middle and inner ears. More work is needed to improve the efficacy of trans-tympanic injections of cisplatin antidotes.

**Trial registration:**

ClinicalTrials.gov, NTC02281006, Registered 3 November 2014.

## Background

Patients with advanced head and neck squamous cell carcinomas (HNSCC) are usually treated with concurrent chemoradiation (CCR) [1]. Cisplatin is a chemotherapeutic agent widely used for these patients and shows better treatment response rates than Carboplatin [2–4]. However, cisplatin is associated with serious dose-limiting side effects such ototoxicity and nephrotoxicity. Cisplatin induces bilateral, symmetric and high-frequency permanent sensorineural hearing loss (SNHL) and thus, greatly alters patients’quality of life [5, 6]. While nephrotoxicity can be prevented by increased saline hydration, controlled diuresis and amifostine administration [7], there are no known preventive treatments available for ototoxicity [7, 8].

Studies have demonstrated that the incidence of ototoxicity following cisplatin therapy varies with the doses administred, age of the patient, cranial irradiation and other factors such as noise exposure and pre-treatment hearing level [6, 9–12]. Cisplatin causes reactive oxygen species to accumulate in the cochlea, which damage the outer hair cells in the organ of Corti [9–13]. Sulfur-containing antioxidants such as sodium thiosulfate can neutralize the cytotoxic effects of cisplatin and could thus theoretically prevent cisplatin-induced ototoxicity [14–19]. However, a major drawback in the use of these molecules when administered systemically is that they decrease cisplatin anti-tumoral efficacy [15, 20] and do not reach easily the inner ear because of the blood-labyrinthic barrier. Recent experimental data on animal models from Berglin et al. indicate that a viscous sodium thiosulfate-hyaluronate gel injected in the middle ear of guinea pigs prior to cisplatin treatment may reduce its ototoxicity without affecting its systemic anti-tumoral effects [18]. To date, only few clinical trials investigated strategies to protect the inner ear from cisplatin-induced ototoxicity with trans-tympanic injections using N-acetylcysteine and dexamethasone, unfortunately reporting only minimal or no effect toward the reduction of cisplatin ototoxicity [21–23]. Thus, we conducted a randomised controlled trial to test the efficacy of a sodium thiosulfate gel administered locally in the middle ear to prevent cisplatin-induced ototoxicity.

## Methods

### Study design

This study is a randomized controlled trial of split body design. For each participant, one randomly selected ear received the intervention while the other ear did not.

### Study setting and population

Participants to the trial were recruited at the radiation therapy department of the CHU de Québec Université Laval (Quebec City, QC, Canada). Eligible participants were patients newly diagnosed with a locally advanced (stage III or IV) squamous cell carcinoma of the mouth, oropharynx, hypopharynx, or larynx scheduled to be treated with concomitant chemoradiation. This treatment combines Intensity Modulated Radiation Therapy (32 to 35 fractions of 2.15 Gy to the tumor) and cisplatin 100 mg/m^2^ on days 1, 22, and 43 after the first radiation fraction. A research nurse presented the trial to potentially eligible patients and obtained a signed informed consent to the investigations to determine final eligibility and to participate in the trial. Personal and medical data were collected including Karnofsky performance status (patients below 70% were not eligible). Final eligibility was determined after an otoscopic examination and an audiometric evaluation. Eligible participants were those with symmetrical hearing (mean of differences between ears at 4 frequencies (3, 4, 6 and 8 kHz) not greater than 10 dB (HL), no difference greater than 10 dB (HL) between air and bone conduction for any ear at 5 frequencies (0.5 to 4 kHz) and normal otoscopic findings. Authorization to use the sodium thiosulfate gel in the trial was obtained from Health Canada (NOL 165615). The trial was approved by the CHU de Québec ethical research committee. An independent Data and Safety Monitoring Committee (DSMC) was established to overview the conduct of the trial comprising experts in biostatistics, audiology and oncology.

### Intervention

A computer generated randomization list with permuted blocks was prepared in advance to assign the ear to be treated for each consecutive eligible and consenting patient. The list was kept secure at the hospital pharmacy department. On the eve of each cisplatin treatment, a pharmacist prepared the gel for the injection by mixing 0.55 ml hyaluronate gel (Healon 10 mg, Abbott Medical Optics Inc) and 0.55 ml of a 25% solution of sodium thiosulfate pentahydrate (Seacalphyx, Seaford Pharmaceuticals Inc). The sodium thiosulfate solution (STS) concentration in the resulting gel was 0.5 M.

Since no previous clinical study used a thiosulfate sodium gel, the concentration of our gel was in part determined with the results of Berglin et al. [18]. In this study, a 0.1 M sodium thiosulfate-hyaluronan gel was injected in the guinea pigs’ middle ears and was found in the scala tympani’s perilymph 1 h after injection. We decided to maximize the concentration of sodium thiosulfate in the hyaluronan gel and to inject a quantity of gel sufficient to only fill the round window area. This was chosen in order to prevent a conductive hearing loss with a full middle ear packing. The highest sodium thiosulfate concentration that could be achieved in order to obtain a stable and homogeneous gel was 0.5 M.

For the procedure, patient’s head was placed tilted at 45 degrees towards the non-treated ear. This position allows the round window niche to be at the lowest point of the middle ear cavity and thus, letting the gel to collect there and be in direct contact with the membrane. After a local anesthesia of the tympanic membrane, the otologist performed a formal otoscopy with a microscope to localize the round window niche, and then deposited 0.1 ml of the gel exactly on it. The gel was introduced in the middle ear precisely via a trans-tympanic injection performed under microscopy, with no extended myringotomy. When the round window niche was not clearly visible through the micro-otoscopy, the gel was injected posteroinferiorly in the middle ear. Patients remained under the otologist’s surveillance until the effects of the anesthesia and the intervention were over.

### Efficacy assessment

To assess the effect of the trial intervention, a complete audiologic evaluation was conducted before chemoradiation therapy and repeated 1 month after the end of chemoradiation. Pure tone air conduction audiograms were performed at frequencies ranging from 0.5 to 14 kHz using an AC-40 clinical audiometer (Interacoustics, calibrated yearly, ANSI S3.6–2004 standards) with inserted earphones (ER-3A, for 0.5 to 8 kHz) and with circumaural earphones (Koss R/80, for 9 to 14 kHz). Bone conduction audiograms were also performed for 5 frequencies ranging from 0.5 to 4 kHz. Sound intensity was measured in decibel hearing level (dB HL). In addition, distortion product otoacoustic emissions (DPOAEs) recordings were performed at 6 frequencies ranging from 1 to 6 kHz using an Echoport ILO-292-USB-II (Otodynamics). The day before proceeding to the second or third trans-tympanic injection, a complete unblinded air conduction audiogram was done for safety monitoring and was not kept blind to the ear assignment. The final audiologic evaluation was conducted by an audiologist blinded to the ear assignment and to the trial records. When the second or third trans-tympanic injection was not performed as scheduled, the complete final audiologic evaluation (including bone conduction and DPOAEs) was conducted before the upcoming chemotherapy.

### Safety monitoring

All side effects and complications possibly related to the intervention occurring during the trial were noted according to the Common Terminology Criteria for Adverse Events (CTCAE) v4.0 [24]. Acute side effects of the trans-tympanic injection were noted by the otologist. A research nurse met with the patient the day after each trans-tympanic injection to note signs and symptoms. An otoscopic examination was performed 2 days following the trans-tympanic injections. The audiologic assessments permitted to measure the ototoxicity of the cisplatin therapy separately for the treated and the control ear using both CTCAE [24] and the American Speech-Language Hearing (ASHA) [25] criteria.

### Data editing

Baseline air conduction data were kept for analysis only when an auditory threshold was observed on both ears for a given frequency. The same approach was used to ensure comparability of data on bone conduction. Presence of baseline DPOAEs was judged on the following two criteria: a signal ≥ − 10 dB (SPL) and a ratio signal to noise ≥6 dB [26]*.* Baseline DPOAEs data were kept for analysis only when otoacoustic emissions were observed on both ears for a given frequency. When there was no air conduction auditory threshold at the final audiologic evaluation at a frequency where there was one at baseline, a numerical value was imputed as the maximum threshold tested plus 5 dB. For high frequencies, the maximum sound intensities tested were respectively 90, 95, 80 and 65 dB (HL) for frequencies 9, 10, 12.5 and 14 kHz. This imputation made it possible to obtain a minimum numerical value for the threshold shift.

### Statistical analysis

The principal outcome measure was the hearing loss defined by the difference between the pure tone air conduction auditory thresholds measured after cisplatin therapy and at baseline, referred to as permanent threshold shift (PTS) in dB (HL). To take into account the dependency between measures recorded at 11 different frequencies, we relied on statistical methods for repeated measurements using a mixed linear model in order to avoid issues associated with multiple comparisons [27, 28]. PTS was the dependent variable while the independent variables were: frequency (11 frequencies ranging from 0.5 to 14 kHz), intervention (treated ear versus control ear), their interaction and the average radiation dose received by the cochlea at the time of the final audiogram. This analysis provided an adjusted estimate of the mean difference of PTS between the treated and the control ears. Similar mixed linear regression models were used for planned secondary analyses. Mean pure tone air conduction auditory PTS difference associated with the intervention for high (9 to 14 kHz), intermediate (4 to 8 kHz) and low (0.5 to 3 kHz) frequencies were calculated. Similarly, mean bone conduction auditory PTS difference was also calculated. The loss of distortion product otoacoustic emissions was compared between the treated and control ears using the same approach.

McNemar tests were used to compare 1) the audiologic toxicity of cisplatin therapy according to CTCAE [24] and ASHA [25] criteria between the treated and the control ears and 2) adverse effects of the trans-tympanic injections.

### Number of participants needed

Data from the first published trial of trans-tympanic injection to prevent cisplatin ototoxicity were used [21] to estimate the number of participants required based on the following assumptions: a mean difference in PTS between treated and control ear of 7.0 dB with the standard deviation of the difference 10.0 dB, a power of 90% and a statistical significance level of 0.045 for the final analysis. An interim analysis was planned when half of the anticipated participants would have completed the follow-up with a test for superiority with two-sided alpha = 0.01. We used the PASS software to estimate the required number of participants: *N* = 25 [29].

## Results

**Fig. 1:**
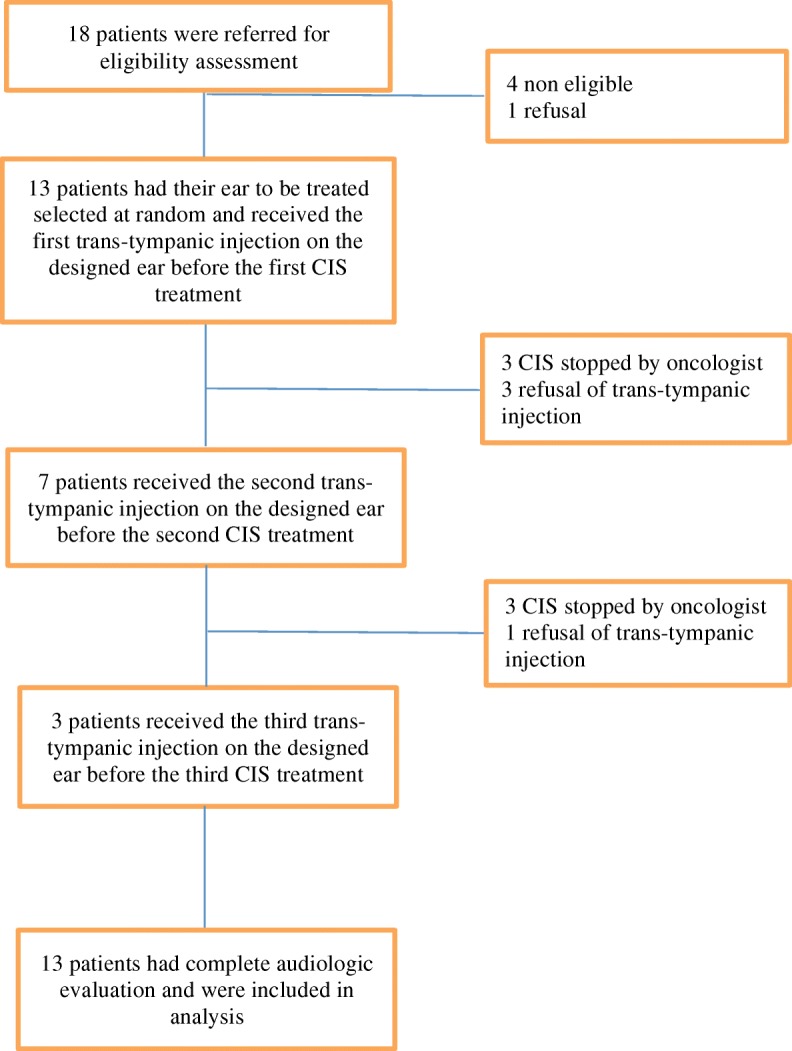
Flow chart

From January 2015 to April 2016, 18 patients were evaluated for eligibility. Of these, 13 were randomized (Table 1). The planned interim analysis was conducted with these 13 participants but no new patients were randomized during the following months and the trial was stopped for poor accrual in June 2016. The decision to end prematurely the study was taken by the research team and was approved by the ethical research committee. Figure 1 (Flow Chart) presents the participants flow. Of the randomized patients, 3 participants received the 3 planned per protocol trans-tympanic injections. Four patients received the first two trans-tympanic injections: three of them had their cisplatin-treatments stopped by their hematologist-oncologist and one of them refused the last injection. Six patients only received the first trans-tympanic injection: three of them had their cisplatin-treatments stopped by their hematologist-oncologist and 3 of them refused the remaining injections.

**Table 1:** Baseline, treatment and intervention characteristics of the 13 trial participants

Age, years, mean SD	58 (8.0)
Sex, male, n %	10 (77)
Smoking, past or current, n %	7 (54)
Weight, kg, mean SD	78 (12)
Education, post-secondary, n %	6 (46)
Karnofsky, 100, n %	13 (100)
Comorbidity, ≥ 1, n %	3 (23)
HNC tumor site	
Oral cavity, n %	4 (31)
Oropharynx, n %	6 (46)
Supraglottic, n %	1 (8)
Unknown, n %	2 (16)
Tumor stage	
III, n %	3 (23)
IVA, n %	7 (54)
IVB, n %	3 (23)
Total Cisplatin dose received at the time of final audiologic evaluation, mg, mean SD	322 (145)
Radiation dose received by the cochlea on the treated side at the time of final audiologic evaluation, grays, mean SD	4.3 (4.5)
Radiation dose received by the cochlea on the control side at the time of final audiologic evaluation, grays, mean SD	7.0 (7.6)
Time between preparation of STS gel and trans-tympanic injection, minutes, mean SD	43.9 (27.5)
Time between trans-tympanic injection and Cisplatin treatment, hours, mean SD	20.6 (0.9)

### PTS outcomes

**Fig. 2:**
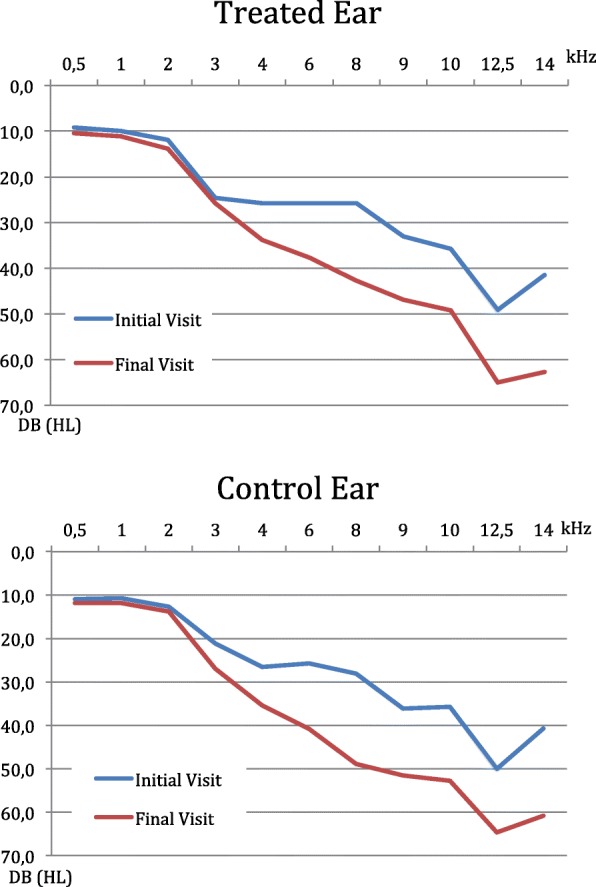
Mean air conduction hearing thresholds at frequencies 0.5 to 14 kHz at baseline and final visits for treated and control ears

**Fig. 3:**
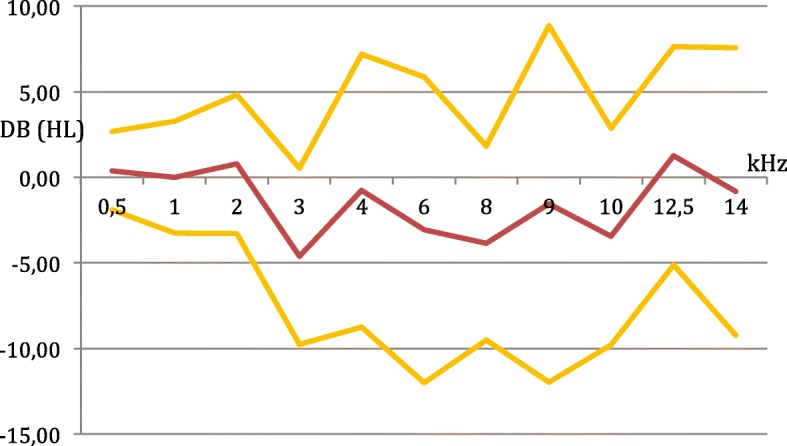
Difference of changes (final minus baseline) in mean air conduction hearing thresholds between treated and control ears: means and 95% confidence intervals at frequencies of 0.5 to 14 kHz

Pure tone thresholds at all frequencies measured at the baseline and final evaluations are presented for the treated and the control ears in Fig. 2 (Mean air conduction hearing thresholds at frequencies 0.5 to 14 kHz at baseline and final visits for treated and control ears). Figure 3 (Difference of changes (final minus baseline) in mean air conduction hearing thresholds between treated and control ears: means and 95% confidence intervals at frequencies 0.5 to 14 kHz) presents the mean difference in PTS between the treated and the control ears at each frequency with 95% confidence intervals. For all frequencies between 3 and 10 kHz the hearing loss was less prononced for the treated ear than for the control. The main outcome analysis using the mixed linear model showed that the average air conduction PTS was 8.50 dB for the treated ears and 9.79 dB for the control ears, for all frequencies (0.5 to 14 kHz). The difference averaged over all frequencies was of 1.30 dB in favor of the treated ears. No statistical difference was found between the average air conduction PTS between the treated and untreated ears (*p* = 0.61).

The difference between the average air conduction PTS at high, intermediate and lower frequencies between the treated and the control ears were respectively 1.09, 2.08 and 1.09 dB, all in favor of the treated ears. However, none of these differences was statistically significant (*p* = 0.81, *p* = 0.57, and *p* = 0.97, respectively). There was no difference (*p* = 0.94) for average bone conduction PTS between the treatred ears (4.33 dB) and the control ear (4,50 dB).

### DPOAEs

Using the mixed linear model, baseline and final DPOAE were compared. The average percentage loss of signal was identical for the treated and the control ears: 22,2% (*p* = 0.99).

### Adverse effects

According to the ASHA criteria [25], ototoxicity was documented in both ears for 7 patients, in the control ear only for 2 patients and in the treated ear only for one patient. For 3 patients, no ototoxicity was observed in either ears (*p* = 0.56). Hearing loss grade of 2 or 3 [24] was found in both ears for 2 patients, in the control ear only for 3 patients, in the treated ear only for none and 8 patients did not present such a hearing loss in any of their ears (*p* = 0.25). Although not significant these differences pointed to less severe hearing loss in the treated ears.

During the trial, 23 trans-tympanic injections were realized. The adverse effects of the trans-tympanic injections were noted after each procedure. In total, three patients reported dizziness and one patient had a vertigo. These side effects both resolved spontaneously after a few minutes. Pain in the middle ear of grade 2 or 3 according to the CTCAE [24], was noted for four patients always on the treated side: for two of them, pain was moderate and for the others, pain was severe.

## Discussion

Our study could not demonstrate statistically significant efficacy of a trans-tympanic injection of a viscous sodium thiosulfate-hyaluronate gel prior to cisplatin therapy to prevent its induced ototoxicity in patients with advanced HNSCC. Slightly better average PTS for the frequencies between 3 and 10 kHz were obtained in treated ears.

Our study demonstrates that trans-tympanic injections of sodium thiosulfate-hyaluronate gel were feasible and safe in terms of both patient compliance and middle and inner ear tolerance (minimal transient adverse side effects). Acute middle ear pain was one of the most frequent adverse effect encountered, but could be explained by the local anesthesia performed in the external ear canal. Furthermore, dizziness and transient mild vertigo could be explained by the gel that could have been colder than the inner ear temperature. Nevertheless, no sensorineural hearing loss was caused by the sodium thiosulfate-hyaluronate gel, also confirming its safety for the inner ear. Similar adverse side effects were also described by Riga et al. in a trial of trans-tympanic infusions of N-acetylcysteine in patients treated with cisplatin-based regimens [21].

For our trans-tympanic injections, the hyaluronic gel was a stabilizer for the sodium thiosulfate and enabled the drug to be highly viscous delaying its elimination. Experimental studies also demonstrated that hyaluronate gel increased the permeability of the round window membrane without toxic effects on the cochlea, allowing the sodium thiosulfate to penetrate in the inner ear [30].

Trans-tympanic injections represent a very attractive approach to prevent cisplatin-ototoxicity since they permit the active agent to reach the inner ear in a higher concentration without decreasing systemic cisplatin efficacy. Moreover, trans-tympanic injections are easily performed in a ENT clinic. An other force of our trial was its study design in which each patient was his own control, thus reducing the number of patients needed and controlling for many potentially confounding factors. One of our limitations was the small number of patients recruited. Another limitation was that for few patients, cisplatin treatments were stopped because of its ototoxic side effects. Accordingly, these patients received a total lower dose of cisplatin. Thus, this could have hampered the possible otoprotective effects of our gel. Moreover, in total, four patients decided to not receive the three transtympanic injections. This could be explained by the fact that these procedures were added to their schedule already filled with their numerous oncologic treatments and follow-ups. Our study schedule was also loaded of medical visits, interventions and audiograms that could have discouraged patients to participate in our study, thus possibly explaining the poor acrual that we had.

To date, three clinical trials, using the same design as ours, investigating trans-tympanic administration of an otoprotective agent to prevent cisplatin ototoxicity have been published. The chemoprotectants studied are agents with antioxidant activity and corticosteroids. Riga et al. [21] assessed trans-tympanic injections of N-acetylcysteine on 20 patients. The only significant effect was observed at 8 k Hz, thresholds changes in the control ears (7.8 dB) were significantly higher than in the ears treated with N-acetylcysteine (0.8 dB). Yoo and his colleagues also experimented N-acetylcysteine trans-tympanic injections on 11 patients [23]. No significant otoprotective effect was found overall, but they observed for two patients a hearing loss dramatically less important at 8 kHz in their treated ear that was still present 3 months after cisplatin treatments. A limitation of this study was that an aqueous solution of 2% L-N-Acetylcysteine was injected, allowing the antioxidant to quickly get through the Eustachian tube shortly after the middle ear infiltration. Marshak et al. [22], investigated the otoprotective effect of dexamethasone trans-tympanic injections on 15 patients. At 8 kHz,, thresholds changes in the control ears were 11.3 dB and 7.4 dB in the treated ears, but the difference was not statistically significant. Results of these three trials are more important on high frequencies, which are the most affected by cisplatin. Unfortunately, main limitations of these previous studies include a small number of patients enrolled and a low total cumulative cisplatin dosage, two majors difficulties that we also encountered in our trial.

Many lines of thought remain from our study. First of all, we decided to inject a small quantity of sodium thiosulfate containing gel in order to prevent full packing of the middle ear with a subsequent conductive hearing loss. The concentration of the gel delivered to the inner ear was 0.5 M. In the study of Berglin et al. [18], on animal model, high concentrations of sodium thiosulfate-hyaluronan gel (0.1 M) delivered in the middle ear were found in the scala tympani’s perilymph. Also, the trans-tympanic injections were done the day before the patients received their cisplatin treatment, allowing some time to the gel to get through the inner ear. The average elapsed time between the injection and the cisplatin treatment was 20.5 h. In the study of Riga et al. [21], the trans-tympanic N-acetylcysteine injections were performed during the hydration procedure preceding intravenous effusion of cisplatin. We chose this timing because it was easier for the patients, according to their treatments’ schedule. Even if patients were asked to remain on lateral decubitus on the controlateral ear and to not swallow nor to blow their nose for 30 min after the injection, for some of them the gel may have poured out by the eustachian tube. The quantity of sodium thiosulfate containing gel injected may not have been sufficient to provide the expected concentration in the cochlea perilymph that would have been otoprotective. Additional studies are required to further determine the optimal dosage and protocol of the sodium thiosulfate-hyaluronate gel**.**

## Conclusions

This randomized controlled trial did not demonstrate a difference between the average PTS of the treated and the control ears. Slightly better average PTS for most of the frequencies tested were obtained in the treated ears. This trial showed that a trans-tympanic injection of a sodium thiosulfate-hyaluronate gel is feasible and innocuous for the middle and inner ears and that further investigations of this innovative gel could be conducted safely. Further research is needed to improve both efficacy of cisplatin therapy and the quality of life of adult and childhood cancer survivors.

## Abbreviations

ASHA: American Speech-Language Hearing Association
CCR: Chemoradiation
CTCAE: Common Terminology Criteria for Adverse Events
DPOAE: Distortion product otoacoustic emissions
DSMC: Data and Safety Monitoring Committee
HNSCC: Head and neck squamous cell carcinomas
PTS: Permanent threshold shift
SNHL: Sensorineural hearing loss
STS: Sodium thiosulfate solution
